# Targeted shock-and-kill HIV-1 gene therapy approach combining CRISPR activation, suicide gene tBid and retargeted adenovirus delivery

**DOI:** 10.1038/s41434-023-00413-1

**Published:** 2023-08-09

**Authors:** Sarah Klinnert, Corinne D. Schenkel, Patrick C. Freitag, Huldrych F. Günthard, Andreas Plückthun, Karin J. Metzner

**Affiliations:** 1 Department of Infectious Diseases and Hospital Epidemiology, University Hospital Zurich, Zurich, Switzerland; 2 Institute of Medical Virology, University of Zurich, Zurich, Switzerland; 3 Life Science Zurich Graduate School, University of Zurich, Zurich, Switzerland; 4 Department of Biochemistry, University of Zurich, Zurich, Switzerland

**Keywords:** Virology, Gene therapy, Genetic vectors, Infectious diseases

## Abstract

Infections with the human immunodeficiency virus type 1 (HIV-1) are incurable due the long-lasting, latent viral reservoir. The shock-and-kill cure approach aims to activate latent proviruses in HIV-1 infected cells and subsequently kill these cells with strategies such as therapeutic vaccines or immune enhancement. Here, we combined the dCas9-VPR CRISPR activation (CRISPRa) system with gRNA-V, the truncated Bid (tBid)-based suicide gene strategy and CD3-retargeted adenovirus (Ad) delivery vectors, in an all-in-one targeted shock-and-kill gene therapy approach to achieve specific elimination of latently HIV-1 infected cells. Simultaneous transduction of latently HIV-1 infected J-Lat 10.6 cells with a CD3-retargeted Ad-CRISPRa-V and Ad-tBid led to a 57.7 ± 17.0% reduction of productively HIV-1 infected cells and 2.4-fold ± 0.25 increase in cell death. The effective activation of latent HIV-1 provirus by Ad-CRISPRa-V was similar to the activation control TNF-α. The strictly HIV-1 dependent and non-leaky killing by tBid could be demonstrated. Furthermore, the high transduction efficiencies of up to 70.8 ± 0.4% by the CD3-retargeting technology in HIV-1 latently infected cell lines was the basis of successful shock-and-kill. This novel targeted shock-and-kill all-in-one gene therapy approach has the potential to safely and effectively eliminate HIV-1 infected cells in a highly HIV-1 and T cell specific manner.

## Introduction

The human immunodeficiency virus type 1 (HIV-1) causes persistent infections, in which cells harboring replication-competent but transcriptionally silent integrated proviruses persist during long-term, suppressive combination antiretroviral therapy (cART) and cause viral rebound after treatment discontinuation [1–4]. cART is a highly effective treatment with numerous benefits for people with HIV (PWH), however, it does not lead to a cure from the infection because it does not affect this HIV-1 latent reservoir [5–7]. Hence, PWH have to take cART life-long, and drug toxicity, drug-drug interactions or drug resistance are just some of the challenges of managing the HIV pandemic, and stress the need for an HIV-1 cure.

One of the most explored cure approaches is the shock-and-kill therapy, which aims to reactivate the latent HIV-1 proviruses and subsequently kill the virus-producing cells (reviewed in [8]).

Pharmacological HIV-1 latency reversal has been extensively tested with latency reversing agents (LRAs) that target cellular pathways and proteins associated with HIV-1 latency to induce proviral transcription (reviewed in [9]). Another shock strategy are CRISPR/dCas9-based activation systems (CRISPRa), which have the advantage of HIV-1 specific latency reversal [10, 11]. In a recent study, we systematically investigated the dCas9-VPR CRISPRa system and demonstrated its potency to reverse HIV-1 latency and identified the optimal guide RNA (gRNA) target region, gRNA-V, in the HIV-1 5’long terminal repeat (LTR) promoter for this system [12].

For the kill phase after HIV-1 reactivation combination with cART is necessary to inhibit replication of the reactivated virus and prevent de novo infection events. Elimination of infected cells in which HIV-1 latency was reversed would occur by viral cytopathic effects and immune responses such as HIV-1 specific cytolytic T cells (CTLs), but it has been shown that these mechanisms do not suffice [13, 14]. Additional kill interventions are crucial and could be provided in the form of therapeutic vaccines, pharmacological agents that revert immune exhaustion, stimulation of CTL responses or enhancement of apoptosis as well as broadly neutralizing antibodies (bnAbs) (reviewed in [8, 15]). Furthermore, suicide gene therapies with different suicide genes have been explored to target productive and latent HIV-1 infection (reviewed in [16]). Our group previously established a suicide gene vector in which the expression of the human pro-apoptotic protein truncated Bid (tBid; BH3 interacting domain death agonist) is highly dependent on the HIV-1 accessory proteins Tat and Rev, leading to a specific, efficient and rapid killing of productively HIV-1 infected cells [17].

To deliver such large DNA complexes, we used adenoviral vectors, retargeted with a trimeric adapter efficiently inhibiting the natural tropism and used for a variety of different receptors and cell types [18, 19]. We implemented the recently developed CD3-specific retargeting adapters, shown to successfully transduce human T cell in various settings [20]. Since CD4^+^ T cells represent the major part of the latent HIV-1 reservoir, we combined this CD3-retargeting technology with the dCas9-VPR activation system, the optimal HIV-1 specific gRNA-V, and the tBid-based suicide gene strategy as a novel shock and kill approach in this study. This novel targeted shock-and-kill all-in-one gene therapy approach has the potential to safely and effectively eliminate HIV-1 infected cells in a specific manner before infectious viral particles are released.

## Results

### HIV-1 shock-and-kill combining CRISPRa, suicide gene tBid and retargeted adenovirus delivery – the model

**Fig. 1:**
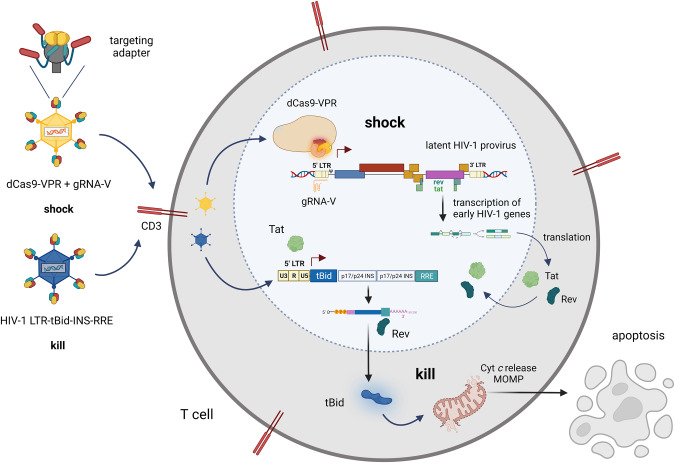
Illustration of the HIV-1 specific targeted shock-and-kill approach with CRISPRa and tBid delivered by CD3-retargeted adenoviruses. Adenovirus vectors ensure effective delivery of the HIV-1 specific CRISPRa shock or HIV-1 specific tBid suicide kill strategies. Moreover, coating of Ads with CD3-retargeting adapters provides a high level of T cell specific delivery. The dCas9-VPR and the HIV-1 specific gRNA-V in the shock Ad-CRISPRa-V activate expression of latent provirus by targeting the HIV-1 LTR promoter. Expression of early genes *tat* and *rev* leads to activation of the tBid suicide vector delivered by the kill Ad-tBid. The Tat protein binds to the HIV-1 LTR promoter driving expression of the tBid suicide gene, and the Rev protein binds to the Rev-responsive element (RRE) in the tBid mRNA enabling export of this mRNA to the cytoplasm. Expression of the tBid protein induces apoptosis very rapidly within 24 h since very low tBid protein amounts suffice to induce release of cytochrome c (Cyt c) and lead to mitochondrial outer membrane permeabilization (MOMP). Figure created with BioRender.com (Figure publication license HO24KAK4FD).

The targeted shock-and-kill strategy that we want to test here consists of the dCas9-VPR and the HIV-1 specific gRNA-V, and the HIV-1 dependent tBid-based suicide vector. We propose a model in which the gRNA-V, which targets the HIV-1 5’LTR −165 to −146 bp from the transcription start site (TSS), will guide the dCas9-VPR CRISPRa system to the HIV-1 promoter and induce transcription of latent HIV-1 provirus. The induced expression of the early HIV-1 genes *tat* and *rev* will subsequently lead to the activation of the tBid-based suicide vector. This occurs by binding of the Tat protein to the HIV-1 5’LTR promoter that drives tBid expression and binding of the Rev protein to the Rev-responsive element (RRE) in the tBid mRNA enabling export of this mRNA to the cytoplasm. In the cytoplasm tBid will be translated and translocated into mitochondria where it induces the release of cytochrome c (Cyt c) and mitochondrial outer membrane permeabilization (MOMP) ultimately resulting in apoptosis (Fig. 1) [21–23].

The tBid-based suicide gene approach fulfills most of the requirements for a functional suicide gene therapy against HIV-1. First, the system is strongly dependent on HIV-1 as shown previously [17]. Second, tBid induces apoptosis in a fast and efficient manner at low tBid protein concentrations before virus particles are released [17, 24]. Third, tBid is not immunogenic since it is a human pro-apoptotic protein. The last requirement is the efficient and specific delivery into HIV-1 target cells, which we aim to address and fulfill here with CD3-retargeted adenoviruses, however not only for the tBid-based suicide kill strategy but also for the CRISPRa shock strategy (Fig. 1).

### HIV-1 latency reversal by dCas9-VPR delivered by CD3 retargeted adenovirus vector

**Fig. 2:**
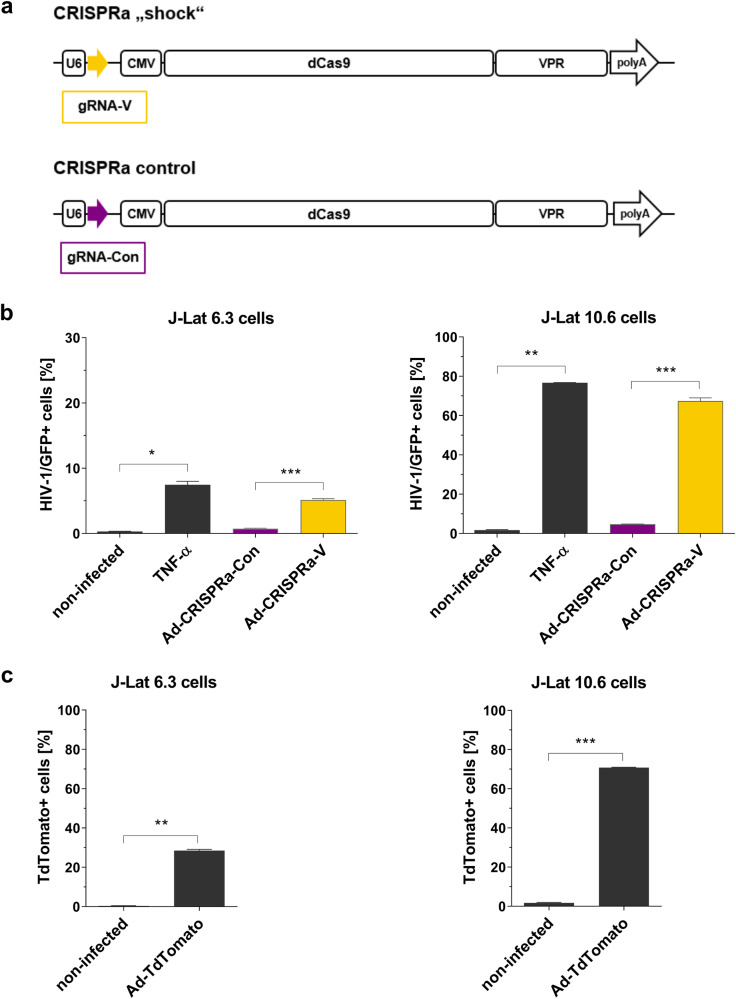
Effective HIV-1 latency reversal by dCas9-VPR and HIV-1 5’LTR specific gRNA-V delivered by CD3-retargeted adenoviruses. **a** CRISPRa constructs cloned into Ad vector. The CRISPRa shock construct contains an expression cassette for the HIV-1 specific gRNA-V, which targets the HIV-1 LTR. Downstream is the dCas9-VPR expression cassette. The CRISPRa control Ad is almost identical, but it contains the control gRNA-Con instead of gRNA-V, which is a random nucleotide sequence that is non-targeting. **b**, **c** 1 × 10^5^ J-Lat 6.3 or 10.6 cells were transduced with 4 × 10^3^ VP (virus particles)/cell of retargeting adapter-coated either (**b**) Ad-CRISPRa-V or Ad-CRISPRa-Con or (**c**) Ad-TdTomato. Ad coating was performed by preincubating Ads with CD3 retargeting adapters in a 50-fold molar excess to adenovirus fiber knob for 1.5 h on ice before addition to cells. Cells were activated with TNF-α [10 ng/ml] as a separate HIV-1 activation control 24 h post transduction. HIV-1 latency reversal and Ad transduction efficiency were measured by flow cytometry 48 h post transduction. **b** HIV-1 latency reversal efficiency by CRISPRa in both cell lines is shown with *n* = 3 + SD as HIV-1/GFP+ cells [%] normalized to the respective Ad transduction efficiencies. Black bars (Untreated, TNF- α) show non-infected cells. **c** Shown is the Ad transduction efficiency in both cell lines as Ad+ cells [%] with *n* = 2 ± SD. Data shown are from three independent experiments. *P < 0.05, **P < 0.002, and ***P < 0.0002 indicate statistical significance between two samples by paired, two-tailed t-test.

Based on our previous findings, we selected one of the most effective HIV-1 specific gRNAs, gRNA-V, in combination with dCas9-VPR and the CD3-retargeted Ad vector to demonstrate efficient delivery of CRISPRa into target cells as well as effective HIV-1 provirus activation (Fig. 2a) [12, 20].

To demonstrate effective HIV-1 latency reversal we selected the cell lines J-Lat 6.3 and J-Lat 10.6, which are latently HIV-1 infected cell lines that harbour a single near full-length latent HIV-1 provirus. The proviruses are replication-incompetent due to a frameshift mutation in *env*. Moreover, since *nef* is replaced by the *gfp* reporter gene, reactivation of the latent provirus by the CD3-retargeted system, termed Ad-CRISPRa-V, could be detected by measuring the expression of GFP via flow cytometry [25].

Cells transduced with the Ad-CRISPRa-V showed high HIV-1 latency reversal in both cell models, whereas the control adenovirus, Ad-CRISPRa-Con did not activate the latent provirus (Fig. 2b). This Ad-CRISPRa-Con control virus is identical to Ad-CRISPRa-V except that it contains the control gRNA, termed gRNA-Con, with random nucleotides. In J-Lat 6.3 cells 5.1 ± 0.3% of transduced cells showed latency reversal, which was comparable to the levels using the activation control TNF-α, showing latency reversal in 7.5 ± 0.7% of cells. The repression of HIV-1 provirus is highest in the J-Lat 6.3 cells compared to all the other J-Lat clones and it is the lowest in the J-Lat 10.6 cells. Hence, in the latter we observed a higher latency reversal of 67.4 ± 2.9% of transduced cells with Ad-CRISPRa-V and this level of activation was also comparable to TNF-α with 76.7 ± 0.2% of cells being HIV-1/GFP-positive upon reactivation.

Using two other control adenovirus, termed Ad-FG-iRFP and Ad-TdTomato, that constitutively express the fluorescent protein iRFP or TdTomato upon transduction of cells, we were able to measure the transduction efficiency with the CD3-retargeting technology. The CD3-retargeting of Ads resulted in a transduction of 28.5 ± 0.9% in J-Lat 6.3 cells and 70.8 ± 0.4% in J-Lat 10.6 cells (Fig. 2c). These high transduction efficiencies of Ad-FG-iRFP with the CD3-retargeting technology were consistent throughout all our experiments as well as in other CD3 expressing T cell lines (Fig. S1). Importantly, we observed that CD3-retargeted compared to non-retargeted Ad-TdTomato showed a 3-fold increase in transduction efficiency in Jurkat T cells, as measured by % of transduced cells (Fig. S2). In summary, the dCas9-VPR and the HIV-1 5’LTR specific gRNA-V was efficiently delivered into T cells by CD3-retargeted Ads and led to a strong activation of latent HIV-1 proviruses.

### Rapid killing of latently HIV-1 infected cells by tBid after latency reversal

**Fig. 3:**
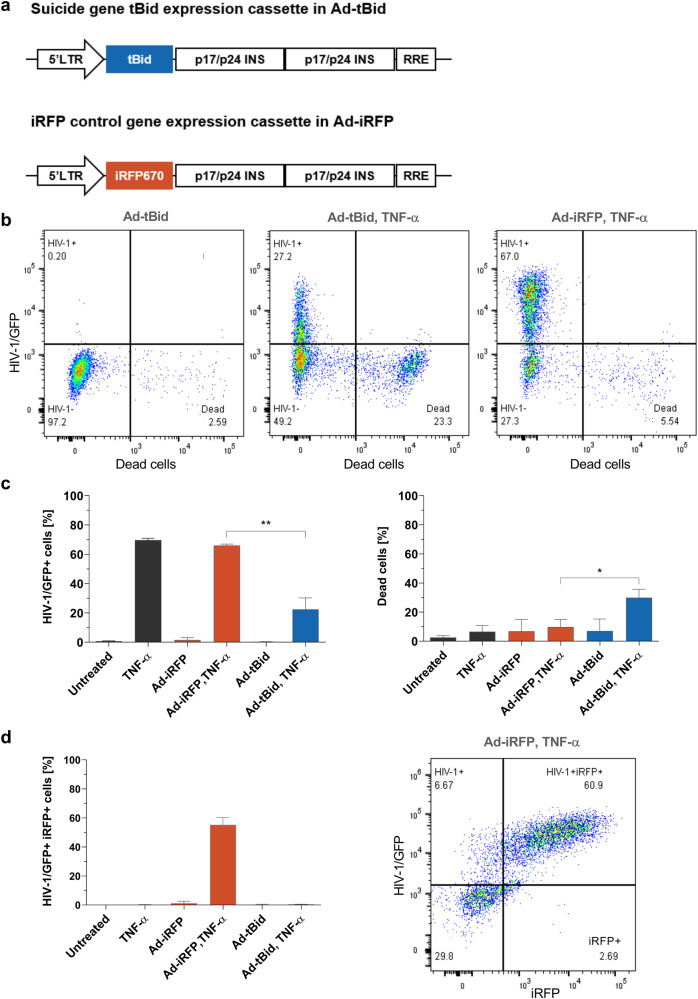
Specific rapid killing of reactivated HIV-1 infected cells by tBid delivered by CD3-retargeted adenoviruses. **a** The suicide and control gene cassettes in the Ad vector are depicted. Gene expression is controlled by the HIV-1 promoter, thus, dependent on HIV-1 Tat. In addition, gene expression is dependent on HIV-1 Rev (RRE, Rev-responsive element) and the two HIV-1 inhibitory sequences (INS) of the HIV-1 gag further enhance Rev dependency. **b**–**d** 1 × 10^5^ J-Lat 10.6 cells were transduced with 4 × 10^3^ VP/cell of retargeting adapter-coated Ad-iRFP or Ad-tBid. Ad coating was performed by preincubating Ads with CD3 retargeting adapters in a 50-fold molar excess over adenovirus fiber knob for 1.5 h on ice before adding the mixture to the cells. HIV-1 latency reversal was achieved by adding TNF-α [10 ng/ml] 24 h post transduction. 48 h post-transduction cells were stained with the dead cell zombie dye and HIV-1 latency reversal as well as suicide vector transgene activation (iRFP or tBid) and cell death were measured by flow cytometry. **b** Exemplary flow cytometry plots showing HIV-1 latency reversal and cell death in Ad-tBid, and TNF-α treated Ad-tBid and Ad-iRFP transduced cells. **c** Shown is HIV-1 latency reversal and cell death with *n* = 3 ± SD, and (**d**) iRFP control transgene activation induced by HIV-1 latency reversal as double-positive HIV-1/GFP+ iRFP+ cells [%] with *n* = 3 ± SD, as well as an exemplary flow cytometry plot of the Ad-iRFP plus TNF-α sample. Black bars (Untreated, TNF- α) show non-infected cells. Data shown from three independent experiments. *P < 0.05 and **P < 0.002 indicate statistical significance between two samples by paired, two-tailed t-test.

Killing of infected cells in which HIV-1 latency was reversed is crucial to eliminate the latent HIV-1 reservoir. Here we combined the CD3-retargeted Ad delivery with a suicide vector that contains the pro-apoptotic protein tBid whose expression is strongly dependent on the HIV-1 regulatory proteins Tat and Rev (Fig. 3a). We focused on the J-Lat 10.6 cell line to show cell killing by the tBid suicide adenovirus, termed Ad-tBid, since successful latency reversal was higher compared to the J-Lat 6.3 cell line (Fig. 2). The corresponding control adenovirus, termed Ad-iRFP, is identical to Ad-tBid except for the substitution of tBid by the iRFP reporter gene (Fig. 2).

Ad transduction with CD3-retargeted Ad-FG-iRFP was again high in these cells (71.7 ± 5.5%) (Fig. S3a). When comparing HIV-1 latency reversal by TNF-α in cells, which were transduced by Ad-tBid or the Ad-iRFP control adenovirus, a significant 66.1 ± 11.8% relative reduction of HIV-1/GFP-positive cells could be observed in Ad-tBid compared to Ad-iRFP transduced cells (Fig. 3b, c). Moreover, at the same time cell death increases 3.6-times ± 1.9 in Ad-tBid transduced cells revealing the rapid elimination of HIV-1 infected cells through cell killing (Fig. 3b, c).

When measuring iRFP in the Ad-iRFP transduced cells treated with TNF-α, an average of 55.2 ± 5.0% of cells were double-positive for HIV-1/GFP and iRFP (Fig. 3d). The iRFP control is expressed via the same Tat/Rev-dependent mechanism as tBid in Ad-tBid, so we can say that killing by tBid should also eliminate up to 55.2 ± 5.0% of cells. Thus, when comparing cell death in the TNF-α treated Ad-iRFP control to the TNF-α treated Ad-tBid cells, an absolute of 43.7 ± 7.7% of HIV-1/GFP+ cells were eliminated (66.1 ± 0.9% vs. 22.4 ± 7.8%, Fig. 3c). Normalizing this absolute number to the Ad transduced and TNF-α activated target population of 55.2 ± 5.0% iRFP-positive cells, the normalized killing effect of this target population, meaning the cells that should contain the tBid suicide vector and show HIV-1 activation by TNF-α, is 78.8 ± 6.6%. Thus, the killing efficacy is about ~80% within 24 h after HIV-1 activation.

Double positive HIV-1/GFP+ dead cells will not appear in our experiment, because with the tBid-based suicide kill strategy, cell death is induced directly after the expression of the HIV-1 early genes tat and rev. The GFP reporter gene in the J-Lat 10.6 provirus is expressed as a late protein, hence its expression is not possible when tBid is activated since cells are killed earlier in a rapid manner [25]. In our previous study, we showed that co-transfection of the tBid-based suicide vector with the HIV-1 full-length clone pNL4-3/GFP also resulted in early cell death induction before virus particles were released and even before GFP was expressed [17].

HIV-1 latency reversal with TNF-α in the Ad-iRFP control showed the same activation level as in cells only treated with TNF-α, but showed no significant induction of cell death as expected (Fig. 3c). In general, in all control settings and cells, which were transduced with CD3-retargeted Ads without a suicide gene, no significant difference in cell death compared to untreated cells could be observed suggesting no increased toxicity by the Ads alone (Fig. 3b, c). Most importantly, no significant increase in cell death between the Ad-tBid only and the other samples demonstrated that there is no detrimental leaky expression of tBid (Fig. 3b, c). The combination of HIV-1 activation by TNF-α and transduction with Ad-tBid was the only one that led to a significant increase in cell death and decrease in HIV-1/GFP-positive cells (Fig. 3c). This HIV-1 specificity of the suicide vector system was also supported by the observation that iRFP expression was exclusively observed in cells that were also positive for HIV-1/GFP in TNF-α treated Ad-iRFP control virus transduced cells (Fig. 3d). No single iRFP-positive cells were detected, since iRFP expression is also highly dependent on HIV-1 Tat and Rev expression, thus HIV-1 activation (Fig. S3b).

In summary, we could show that the tBid or iRFP transgene vectors delivered by CD3-retargeted Ads were exclusively activated when HIV-1 was activated and that these cells were rapidly eliminated by the induction of cell death.

### Combined HIV-1 specific shock-and-kill eliminates HIV-1 reactivated cells

After successfully testing the single components of our targeted shock-and-kill approach, we combined the targeted shock Ad-CRISPRa-V with the targeted kill Ad-tBid and performed a co-transduction of J-Lat 10.6 cells.

**Fig. 4:**
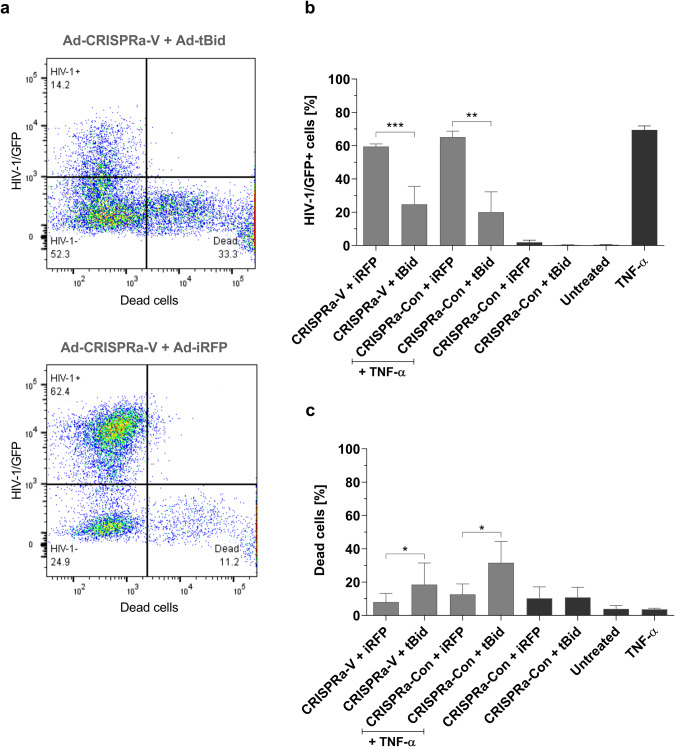
HIV-1 specific CRISPRa shock and tBid suicide gene kill delivered by CD3-retargeted adenoviruses. 1 × 10^5^ J-Lat 10.6 cells were transduced with a total of 8 × 10^3^ VP/cell and a 1:1 ratio of two different retargeted Ads at the same time, either Ad-tBid or Ad-iRFP and Ad-CRISPRa-V or Ad-CRISPRa-Con. Ad coating was performed by preincubating Ads with CD3 retargeting adapters in a 50-fold molar excess over adenovirus fiber knob for 1.5 h on ice before addition to cells. HIV-1 latency reversal in the CRISPRa-Con controls was achieved by adding TNF-α [10 ng/ml] 24 h post transduction. At 48 h post-transduction cells were stained with the dead cell zombie dye and measured by flow cytometry. **a** Exemplary flow cytometry plots showing HIV-1 latency reversal and cell death in Ad-CRISPRa-V, and Ad-tBid or Ad-iRFP, co-transduced cells. **b**, **c** Shown are HIV-1 latency reversal and cell death in the dual transduced cells with *n* = 3 ± SD. Black bars (Untreated, TNF- α) show non-infected cells. Data shown from three independent experiments. *P < 0.05, **P < 0.002 and ***P < 0.0002 indicate statistical significance between two samples by paired, two-tailed t-test.

Ad-CRISPRa-V together with Ad-tBid revealed a profound relative reduction of the HIV-1/GFP-positive cell population by 57.7 ± 17.0% compared to cells co-transduced with Ad-iRFP instead of Ad-tBid (Fig. 4a, b). The absolute reduction of HIV-1/GFP-positive cells was on average 34.8 ± 11.1% and the normalized killing effect of the target population, normalized to the 45.5 ± 3.3% HIV-1/GFP+iRFP+ double-positive population, was 74.1 ± 19.2% (Fig. 4b and S4a). This result illustrates the activation of tBid expression in the suicide vector through the Ad-CRISPRa-V induced HIV-1 latency reversal leading to killing of these cells (Fig. 1). In addition, this was reflected through the 2.4-fold ± 0.9 higher cell death in cells co-transduced with Ad-CRISPRa-V and Ad-tBid (Fig. 4a, c).

HIV-1 latency reversal by TNF-α with Ad-tBid showed a 69.8 ± 17.3% relative reduction of the HIV-1-positive cell population and a 2.4-fold ±0.25 increase in cell death compared to TNF-α and Ad-iRFP control (Fig. 4). The absolute reduction of HIV-1/GFP-positive cells was on average 45.1 ± 9.1% and the normalized killing effect of the target population, normalized to the 47.2 ± 1.3% HIV-1/GFP+iRFP+ double-positive population, was 89.3 ± 12.4% (Fig. 4b and S4a). Hence, the combined Ad-CRISPRa-V shock and Ad-tBid kill was similar effective as TNF-α.

Approximately 24.7 ± 10.8% of the HIV-1 + /GFP+ live cell population remained alive and were not killed in the Ad-CRISPRa-V and Ad-tBid co-transduced sample (Fig. 4b). This cell population represents the cells that were only transduced with Ad-CRISPRa-V but not with Ad-tBid. Due to the co-infection not all cells were transduced with both adenoviruses, but there were two additional cell populations transduced with either only one or the other Ad. This could be confirmed with the Ad-iRFP, which expresses iRFP in the same Tat/Rev-dependent manner as tBid is expressed by Ad-tBid. In the Ad-CRISPRa-V and Ad-iRFP co-transduced cells, 45.5 ± 3.3% of the cells were HIV-1/GFP+iRFP+ double-positive meaning that they were transduced by both adenoviruses (Fig. S4a). At the same time, 15.0 ± 2.3% of cells were HIV-1/GFP+ single-positive, representing the cell population that was transduced by Ad-CRISPRa-V only (Fig. S4d).

The uniqueness of our targeted shock-and-kill approach lies in the HIV-1 specificity of both components, which could be verified especially for the tBid-based kill strategy. The HIV-1 specific gRNA-V and dCas9-VPR induced iRFP expression in the control and this was exclusively restricted to HIV-1-positive cells, as no single iRFP-positive cells could be detected (Fig. S4b, c). HIV-1 latency reversal with TNF-α exhibited the same effect confirming that iRFP expression is only induced after HIV-1 latency reversal. In contrast, Ad-CRISPRa-Con showed no activation of the respective kill strategy transgenes, iRFP control or tBid, since there is no HIV-1 activation with the control gRNA (Fig. 4 and S4a).

Lastly, the combination of both targeted shock-and-kill components with each other appeared to be safe since no expression of the kill transgenes by the LTR targeting CRISPRa was detected in a co-transduction experiment in HIV-1 uninfected Jurkat cells (Fig. S5).

## Discussion

The shock-and-kill approach is one of the most researched and clinically advanced HIV-1 cure approaches. Nevertheless, studies to date mainly focus on the shock aspect and very few show successful combination with “kill” strategies. Here, we applied the CRISPR activation (CRISPRa) shock strategy together with a tBid-based suicide gene kill strategy and demonstrate the effective elimination of latently HIV-1 infected cells in the J-Lat HIV-1 latency model. Moreover, delivery of both strategies through CD3-retargeted adenoviral vectors transforms this approach into a novel targeted shock-and-kill therapy with a very high degree of HIV-1 and T cell specificity.

CRISPRa systems are a novel strategy to reverse HIV-1 latency and exhibit a higher reactivation potency compared to most LRAs in vitro [26–29]. We have previously shown that the dCas9-VPR system and gRNA-V in particular have a high HIV-1 activation potency in different latently infected cell line models and it is also capable of reactivating replication-competent provirus [12]. Cells of the two latently HIV-1 infected cell lines used in this study each harbour a single near full-length, replication-incompetent HIV-1 provirus that is predominantly transcriptionally silent. Proviral repression levels are higher in J-Lat 6.3 cells compared to J-Lat 10.6 cells [30, 31]. In line with previous studies, we show a strong HIV-1 reactivation in J-Lat 10.6 and in J-Lat 6.3 cells by our new strategy that is as effective as TNF-α, which is a very strong HIV-1 activator in these cells [12, 32, 33].

Here, we combined dCas9-VPR and gRNA-V with a tBid-based suicide gene strategy. tBid is a death agonist belonging to the pro-apoptotic Bcl-2 family and tBid has been investigated as a therapeutic or suicide gene in cancer therapy, also in combination with adenoviral vectors [34–36]. In the context of latent HIV-1 infection, expression of anti-apoptotic proteins, e.g. Bcl-2, is elevated whereas pro-apoptotic proteins are inhibited favoring cell survival of latently infected cells. Hence, kill approaches that manipulate the cell survival or apoptosis pathway with pro-apoptotic compounds such as Bcl-2 antagonists are of interest (reviewed in [37]). The induction of apoptosis by tBid and its kinetics have previously been demonstrated by measuring the expression of apoptosis markers such as Annexin-V, caspases 3, 8 and 9, or by performing cell viability assays [34, 35]. Cell killing by tBid happens very rapidly namely in the range of 50–70 s when it is applied as a compound in subnanomolar concentrations [24]. And doxycyclin-dependent induction of tBid expression showed killing of up to 98% of cells within 24 h [38]. Our group designed an HIV-1 LTR-based and HIV-1 Tat- and Rev-dependent tBid expression cassette and demonstrated its applicability against productively HIV-1 infected cells. In the presence of both viral proteins, Tat and Rev, cell death was induced very efficiently and rapidly within 24 h [17]. Here, we confirm the tBid induced killing in latently infected J-Lat 10.6 cells after HIV-1 latency is reversed with TNF-α, and furthermore show that elimination of HIV-1 infected cells is equally effective when the tBid-based suicide gene kill strategy is combined with the CRISPRa shock strategy.

In addition to its effectivity, our shock-and-kill approach offers a high level of HIV-1 specificity. The most prominent advantage of CRISPRa systems over pharmacological shock with LRAs is that they reactivate latent HIV-1 proviruses in an HIV-1 specific manner. Specificity is crucial to minimize side effects and toxicity as these were observed in clinical trials with HIV-1 unspecific LRAs that target cellular proteins and pathways [10]. CRISPRa systems and gRNA-V have shown no detrimental off-target activation of cellular genes or dysregulation of transcription, cell cycle progression or cell viability in vitro [28, 39, 40]. A study by J.F.S. Mann et al. stressed the advantages of HIV-1 specific latency reversal and presented an HIV-1 specific “LRA” based on HIV virus-like particles [41]. Also the kill intervention in our approach is HIV-1 specific, because it contains the HIV-1 5’LTR as a promoter, the Rev-response element and two HIV-1 inhibitory sequences (INS) of the HIV-1 *gag* gene. All elements together make the expression of tBid strictly dependent on the expression of both HIV-1 accessory proteins Tat and Rev as previously shown by us and others [17, 42]. This strict dependency is confirmed in our findings resulting in a tightly controlled system.

Killing by a suicide gene strategy as proposed here does not depend on a strong HIV-1 antigen expression and presentation, thus it could circumvent the need of sufficient viral antigen expression as for CD8 + T cell-mediated immune-based kill interventions [14]. Furthermore, cells that harbor defective proviruses capable of expressing the early proteins Tat and Rev, which constitute ~6.5% of defective proviruses, will also be eliminated with our tBid-based kill strategy [43]. Since defective proviruses and especially Tat may contribute to HIV-1 pathogenicity and HIV-1 associated chronic comorbidities, the elimination of defective proviruses needs to be considered [44].

The main limitation of our study is that we show the efficacy of our approach in HIV-1 latency cell line models that do not harbor replication-competent provirus. Despite the fact that the J-Lat cells contain wild-type Tat and Rev which are essential for our system, it needs to be further tested in a primary cell model or in patient-derived cells. The results of our study are however encouraging, because LRAs that lead to a strong latency reversal in the J-Lat cells were often also effective in latently infected CD4^+^ T cells ex vivo [32]. Furthermore, it is unclear whether CRISPRa system can overcome the limitation of LRAs of inducing transcriptional elongation and splicing in primary cells [45]. The combination of CRISPRa systems with a selective activator of the RasGRP1-Raf-ERK1/2 pathway might be a possibility to loosen the elongation block in resting CD4 + T cells to enable sufficient expression of Tat and Rev for our kill strategy [46].

An additional level of specificity is added to our approach by our delivery platform. Delivery of our optimized shock-and-kill components with the human adenovirus serotype 5 vector (HAd-C5) using a CD3-retargeting technology enabled high transduction efficiencies of 70.8 ± 0.4% to 71.7 ± 5.5% in J-Lat 10.6, and 28.5 ± 0.9% to 69.1 ± 8.2% in J-Lat 6.3 cells.

The major obstacle for clinical application of CRISPRa systems is the effective and safe in vivo delivery. Both adenovirus and lentivirus vectors are suitable for spCas9-based therapies and especially CRISPRa systems, whose transgene sizes range from ~5.2 to 7.5 kb, depending on the system [27, 47]. Ad vectors offer non-integrating delivery of transgenes with increased safety; however, the low transduction efficiency especially of T cells by non-targeted unmodified Ad thus far had been a major limitation for their use [48, 49].

Specific targeting of T-cells or HIV-1 infected cells for HIV-1 therapy is a major goal that has already been pursued in many different ways, for instance with a CD7-specific single-chain variable fragment antibody, CD4-specific DARPins or HIV-1 gp140 [50–52]. By deploying the CD3-retargeting technology, delivery into T cells, the main target of HIV-1 therapy, is much more effective and occurs in a more specific manner getting one step closer to the ideal gene therapy delivery vector that combines a large packaging capacity, effective transduction, high safety margin, and receptor-specific transduction [20]. Lastly, in our previous study we showed that Ad adapter retargeting can be applied on helper-dependent Ads, which have a delivery capacity of 35 kb, and that it can be combined with a capsid-covering protein coat [53, 54]. For further in vivo studies we plan to apply both, helper-dependent Ads and immune shielding, to deliver both our strategies in one Ad and prevent immune recognition.

## Conclusion and outlook

In summary, our novel targeted shock-and-kill HIV-1 gene therapy approach presented here has the potential to safely and effectively eliminate HIV-1 infected cells in a highly HIV-1 specific manner before infectious virus particles are released.

Furthermore, this approach comprises a high degree of flexibility and customizability: (1) Multiple HIV-1 specific gRNAs can be used to cover HIV-1 proviral diversity to target all proviruses present in a patient. (2) Both shock-and-kill components could be delivered in a gutless adenoviral vector to have both effectors in the same cell. (3) Cell and tissue-specific promoters could restrict the expression of the Ad cargo further. (4) Ad immune shielding can be achieved with a capsid-covering shield to prevent immune recognition in vivo [53]. (4) Different receptor-targeting modules, *e.g*. specific for CD4, or even combinations can be applied to further narrow down specificity to HIV-1 infected cells, and targeting could be expanded to include other cell types that contribute to the latent HIV-1 reservoir such as monocytes/macrophages. The holy grail for HIV-1 eradication, and the HIV-1 specific targeting and elimination with our targeted shock-and-kill approach, would entail the use of adapters that target a biomarker of the latent HIV-1 reservoir, since adapters against essentially any surface protein can be made [19]. The cell surface protein CD32a, for instance, was proposed as such a HIV-1 latency marker [55, 56]; however, several other studies found contradicting results, whereby the search for reliable and specific HIV latency biomarkers in resting CD4^+^ T cells is ongoing [57–62].

## Materials and methods

### Plasmids and cloning

SP-dCas9-VPR was a gift from G. Church (Addgene plasmid # 63798, Watertown, MA, USA), and pSPgRNA was a gift from C. Gersbach (Addgene plasmid # 47108) [63, 64]. Cloning of the HIV-1 5’LTR specific gRNA-V into pSPgRNA was performed as previously described and cloning of the random nucleotides control gRNA, gRNA-Con (gRNA-Con fw, gRNA-Con rev) was performed in the same way [12]. The gRNA-V and gRNA-Con expression cassette of the pSPgRNA plasmid was amplified from the plasmid using the primers dCasVPR_U6gRNA_fw and U6gRNA_dCas9VPR_rev (Table S1). The amplicon contained overlaps to the MfeI restriction site in the SP-dCas9-VPR plasmid, situated upstream of the dCas9-VPR expression cassette, and was cloned into the MfeI digested plasmid using the In-Fusion^®^ HD Cloning Kit (Takara Bio Europe., Saint-Germain-en-Laye, France) as described in the manufacturer’s instructions. The resulting plasmids contained the gRNA-V, or gRNA-Con, and dCas9-VPR expression cassettes. Both were amplified together in one amplicon from the plasmid using the primers PS1_CRISPRa_fw and PS1_CRISPRa_rev. The amplicon was cloned into the KpnI digested pShuttle (PS-1) vector from the AdEasy adenoviral vector system by Agilent Technologies (Santa Clara, CA, USA) using In-Fusion cloning.

The tBid suicide gene cassette from the vector pLtBid(INS)_2_R, was cloned into the KpnI digested PS-1 vector with Gibson Assembly (NEB Master Mix, E2611, Ipswich, MA, USA) using the primers GibA_tBid-PS1_fw and GibA_tBid-PS1_rev [17]. The expression cassettes in the PS-1 vector had to be modified prior to cloning into the pAdEasy-1_HVR7 adenoviral delivery vectors, because undesired recombination occurred during adenovirus production most likely caused by the homologous LTRs. The 3’LTR was removed by digesting the PS-1_tBid vector with XhoI and a synthetic polyA signal was cloned in by In-Fusion cloning with the primers InFusion_polyA_fw and InFusion_polyA_rev. The resulting PS-1_tBid_polyA vector was used to replace tBid with the reporter gene iRFP670. The PS-1_tBid_polyA plasmid was digested with NheI and BlpI to remove tBid and iRFP670 was cloned in with Gibson Assembly using the primers GibsAss_iRFP670_fwd and GibsAss_iRFP670_rev. The control vector was termed PS-1 iRFP control.

Cloning of the CRISPRa and the modified tBid/iRFP expression cassettes from the respective PS-1 vectors into pAdEasy-1_HVR7 adenoviral vector was performed according to the manufacturer’s instructions. The pAdEasy-1_HVR7 adenoviral vector is a modified version of the original pAdEasy-1, which reduces liver infection [53]. All intermediate and final plasmid sequences were confirmed by sequencing. HAdV5^HVR7^ recombinant adenoviruses with the respective CRISPRa and tBid/iRFP expression cassettes were produced by Vector Biolabs (Malvern, PA, USA). All cloning primers are listed in Supplementary Table S1.

### Cell culture

The cell lines used in this study were obtained through the NIH AIDS Reagent Program, Division of AIDS, NIAID, NIH: Jurkat Clone E6-1 from Dr. Arthur Weiss, J-Lat full length cells clone 6.3 and clone 10.6 from E. Verdin [25, 65]. Jurkat and J-Lat cells were maintained in RPMI-1640 medium supplemented with 2 mM L-glutamine, 10% heat-inactivated fetal bovine serum (FBS) and 1% penicillin/streptomycin (P/S) at 37 °C and 5% CO_2_.

### Adenovirus retargeting and transduction

Adenoviruses (Ads) containing either the CRISPRa-V, -Con, tBid or iRFP expression vectors were preincubated with CD3 retargeting adapters in a 50-fold molar excess over adenoviral fiber knob for 1.5 h on ice in PBS. The reporter adenoviruses Ad-FG-iRFP and Ad-TdTomato containing the reporter genes iRFP670 and TdTomato respectively, which are both under the control of a CMV promoter, were used as control viruses to measure the transduction efficiency achieved with CD3-retargeting. Production and purification of CD3 retargeting adapters was performed as previously described [20]. 1 × 10^5^ J-Lat 6.3 or J-Lat 10.6 cells were transduced with the adapter-coated Ads with 8 × 10^3^ virus particles (VP)/cell. 24 h post transduction the virus-containing supernatant was replaced with fresh medium and HIV-1 activation controls were activated with 10 ng/ml TNF-α (tumor necrosis factor alpha) and 48 h post transduction the readout was performed by flow cytometry.

### Flow cytometry

At 48 h post Ad transduction cells were washed twice with PBS, fixed with 2% paraformaldehyde and fluorophore expression was measured in a minimum of 10.000 events per sample with a BD LSR II Fortessa 4 L (BD Biosciences, Franklin Lakes, NJ, USA). For the detection of cell death, cells were washed twice with PBS, stained with Zombie NIR™ Fixable Viability Kit (BioLegend, San Diego, CA, USA) according to the manufacturer’s protocol, washed again twice with PBS and fixed. HIV-1/GFP-, iRFP-, TdTomato-positive or dying/zombie dye-positive cells were analyzed with FlowJo software v10.0.8.

### Statistical analysis

The data are presented as the mean and standard deviation (SD) and statistical analysis was performed using Prism 9 software (Graph Pad, San Diego, CA, USA). Every experiment was independently performed 2-3 times using duplicates or triplicates as stated in each result section, and no samples were excluded. The raw data were first subjected to a normality and lognormality test to determine its distribution, and if it was lognormal distributed the statistical test was performed on log10 transformed data. Paired, two-tailed t-test was performed between two indicated samples to evaluate the statistical significance of the treatments. A result of *P* < 0.05 was considered to be statistically significant. *P* < 0.05 is indicated with *, *P* < 0.002 with ** and *P* < 0.0002 with ***.

### Supplementary information

Klinnert_et_al_supplementary_material

## Data Availability

The data supporting the findings of this study are available within the article and its supplementary materials.
