# Supplementary material for: Targeted shock-and-kill HIV-1 gene therapy approach combining CRISPR activation, suicide gene tBid and retargeted adenovirus delivery
Source: Gene Ther. 2023 Aug 9;31(3-4):74–84. doi: 10.1038/s41434-023-00413-1 (PMC10940146; doi:10.1038/s41434-023-00413-1)
Supplement: Supplementary file 1 — Klinnert_et_al_supplementary_material [file 41434_2023_413_MOESM1_ESM.docx]

Targeted shock-and-kill HIV-1 gene therapy approach combining CRISPR activation, suicide gene tBid and retargeted adenovirus delivery

Sarah Klinnert^1,2,3^, Corinne D. Schenkel^1,2^, Patrick C. Freitag^3,4^, Huldrych F. Günthard^1,2^, Andreas Plückthun^4^, and Karin J.Metzner^1,2,*^

#### Supplementary Material

Table S1: Oligonucleotides (Microsynth, Switzerland)

| **Primer name** | **Sequence (5’-3’)** |
| --- | --- |
| gRNA-Con fw | CACCATGTACACTCGGCGCAAAGT |
| gRNA-Con rev | AAACACTTTGCG CCGAGTGTACATC |
| dCasVPR_U6gRNA_fw | GCTTGACCGACAATTCGGTACCGAGGGCCTATTTCCCATGATTCC |
| U6gRNA_dCas9VPR_rev | ATTCTTCATGCAATTGGAAAAAAAGCACCGACTCG |
| PS1_CRISPRa_fw | ATAGCGCGTAATACTGGAGGGCCTATTTCCCATGATTCC |
| PS1_CRISPRa_rev | ACTCGAGGCG GCCGCGGAAGCGGCCTTAGTTATTCAGCG |
| GibA_tBid-PS1_fw | TACTCATAGCGCGTAATACTGGTACCAGTTCTACTTACACCAGGAAAGG |
| GibA_tBid-PS1_rev | TCTAGACTCGAGGCGGCCGCGGTACTCACTGCAACCTCTACCTC |
| InFusion_polyA_fw | CTAGAGCGGCCTCGAAATAAAAGATCTTTATTTTCATTAGATCTGTG |
| InFusion_polyA_rev | TATCTCTAGACTCGACTCTAGACACACAAAAAACCAACAC |
| GibsAss_iRFP670_fwd | CCGAACAGGGACTTGAAAGCGAAAGGCGGCCGCATGGCC AGAAAGGTGGAC |
| GibsAss_iRFP670_rev | CCGCGCGCTTCAGCAAGCCGAGTCCCGATCGT CATCATCTCTGGTGGTGAG |


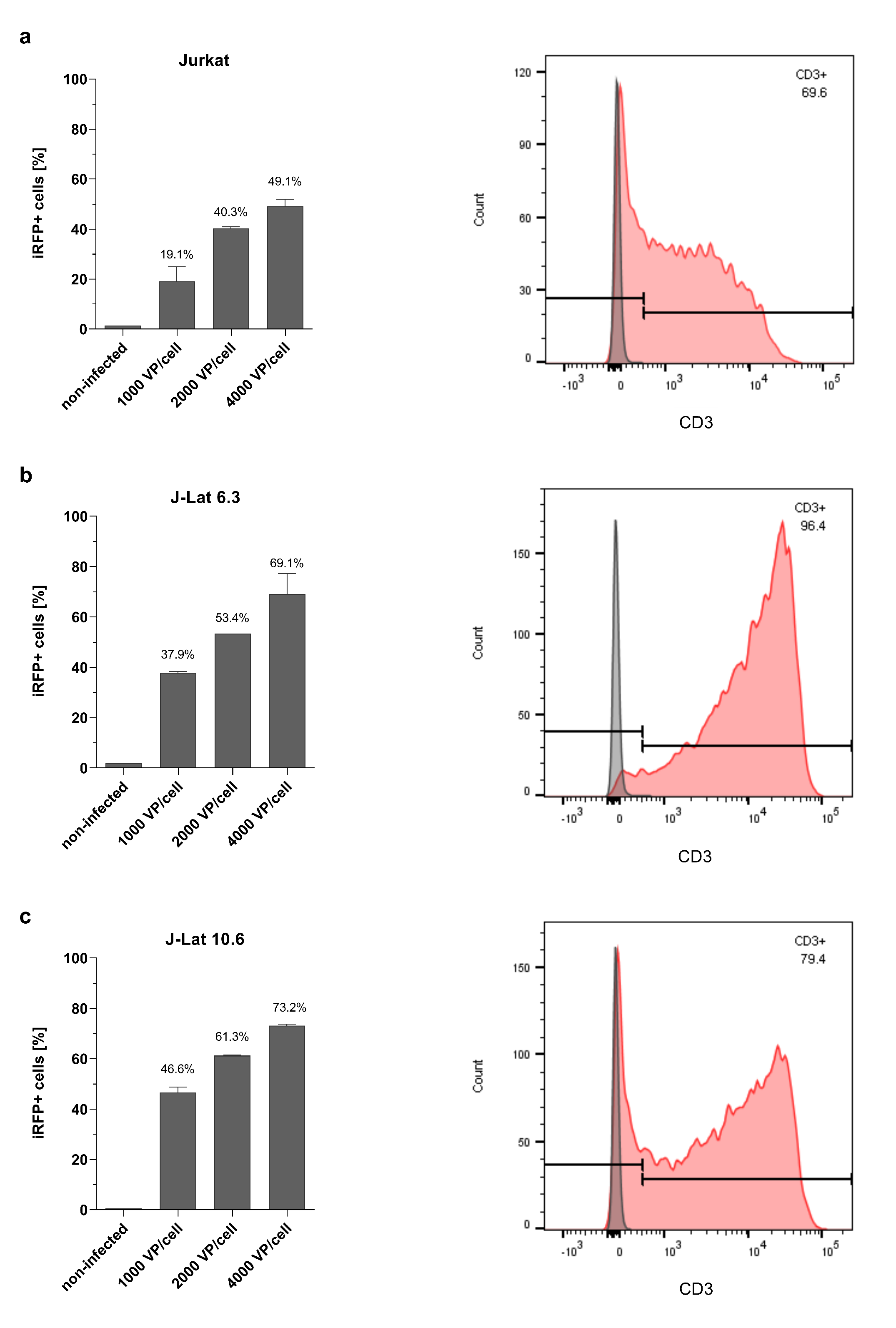


Supplementary figure S1: Adenovirus transduction with CD3-retargeting adapters of various CD3-expressing T cell lines. (a) Jurkat cells, (b) J-Lat 6.3 cells and (c) J-Lat 10.6 cells were stained with a CD3 antibody (CD3 (HIT3a clone)-APC, Biolegend 300312) for 30 min at 4°C to determine CD3 expression levels. Prior to and after staining, cells were washed twice with PBS and measured by flow cytometry. Ad transduction was assessed by transducing 1x10^5^ cells with 1x10^3^, 2x10^3^ or 4x10^3^ VP/cell of CD3-retargeted Ad-FG-iRFP. Ad coating was performed by preincubating Ads with CD3-retargeting adapters in a 50-fold molar excess over adenovirus fiber knob for 1.5 h on ice before addition to cells. Flow cytometry was performed at 48 h post-transduction measuring iRFP expression to asses Ad transduction efficiency and is shown as iRFP+cells [%] ±SD, n=2.


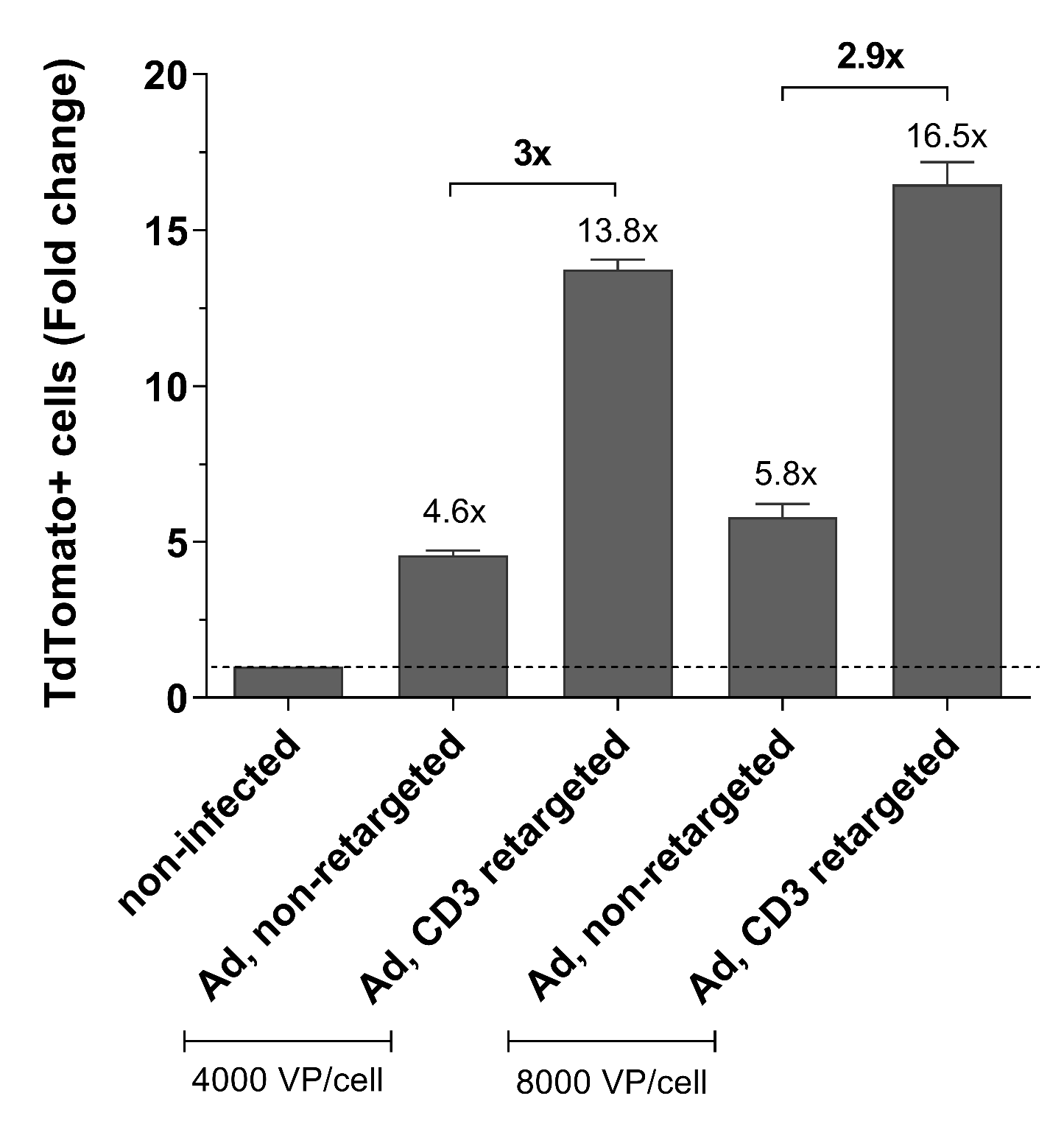


Supplementary figure S2: Effect of CD3-retargeting on Ad transduction efficiency**.** 1x10^5^ Jurkat cells were transduced with 4x10^3^ or 8x10^3^ VP/cell of CD3-retargeted or non-retargeted Ad-TdTomato. Ad coating was performed by preincubating Ads with CD3-retargeting adapters in a 50-fold molar excess over adenovirus fiber knob for 1.5 h on ice before addition to cells. Ad transduction efficiency was measured by flow cytometry 48 h post-transduction. Shown is the fold change of Ad transduction efficiency over untreated cells as TdTomato+ cells (fold change) ±SD with n=2.


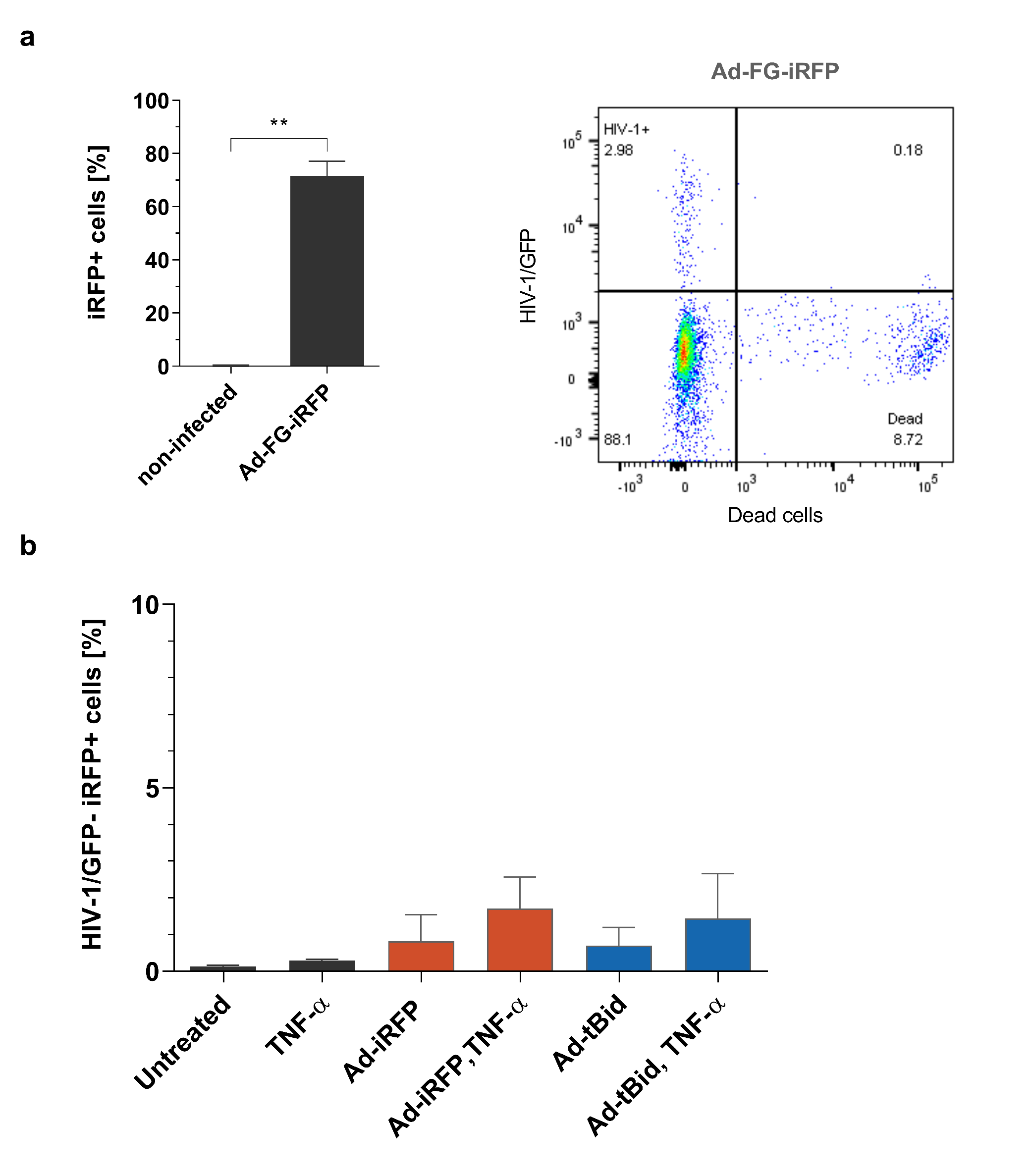


Supplementary figure S3: HIV-1 specific killing by Ad-tBid in latently infected J-Lat 10.6 cells**.** 1x10^5^ J-Lat 10.6 cells were transduced with 4x10^3^ VP/cell of retargeting adapter-coated Ad-iRFP, Ad-tBid or Ad-FG-iRFP. Ad coating was performed by preincubating Ads with CD3-retargeting adapters in a 50-fold molar excess over adenovirus fiber knob for 1.5 h on ice before addition to cells. HIV-1 latency reversal was achieved by adding TNF-α [10 ng/ml] 24 h post transduction. 48 h post-transduction cells were stained with the dead cell zombie dye and HIV‑1 latency reversal as well as suicide construct transgene activation (iRFP or tBid) and cell death were measured by flow cytometry. (a) Shown is the Ad transduction efficiency ±SD with the Ad-FG-iRFP reporter virus as iRFP+ cells with n=2 from independent experiments, as well as an exemplary flow cytometry plot of HIV-1 latency reversal and cell death of the same sample. *P<0.033 and **P<0.002 indicate statistical significance between two samples by paired, two-tailed t-test.(b) No leaky expression of the iRFP transgene is observed shown as HIV-1/GFP negative iRFP+ single-positive cells [%] ±SD with n=3. Black bars (Untreated, TNF- α) show non-infected cells. Data shown from three independent experiments.

**
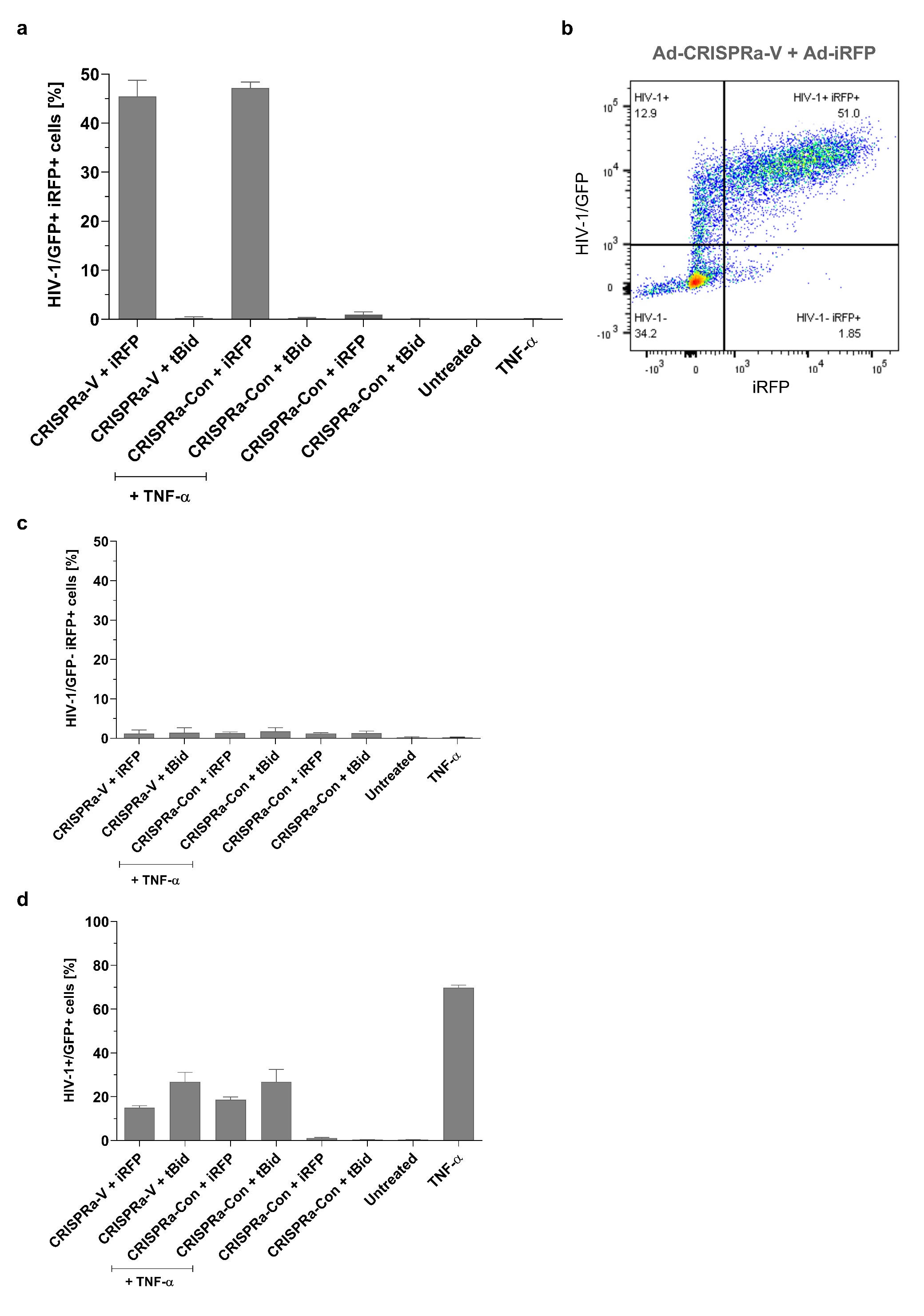
**

Supplementary figure S4: Targeted shock and kill with CD3-retargeted Ads in latently infected J-Lat 10.6 cells**.** 1x10^5^ J-Lat 10.6 cells were co-transduced with a total of 8x10^3^ VP/cell with two different retargeted Ads at the same time, either Ad-tBid or Ad-iRFP and Ad-CRISPRa-V or Ad-CRISPRa-Con. Ad coating was performed by preincubating Ads with CD3-retargeting adapters in a 50-fold molar excess over adenovirus fiber knob for 1.5 h on ice before addition to cells. HIV-1 latency reversal in the CRISPRa-Con and Cell controls was achieved by adding TNF-α [10 ng/ml] 24 h post transduction. At 48 h post-transduction cells were stained with the dead cell zombie dye and measured by flow cytometry. (a) Shown are simultaneous HIV-1 latency reversal and iRFP transgene activation as HIV-1+/GFP+ iRFP+ double-positive cells [%] ±SD with n=3 from three independent experiments.(b) Exemplary flow cytometry plots of HIV-1+/GFP+ iRFP+ double-positive cells in Ad-CRISPRa-V and Ad-iRFP co-transduced cells. (c) No leaky expression of the iRFP transgene is observed shown as HIV‑1/GFP negative iRFP+ single-positive cells [%] ±SD with n=3 from three independent experiments. (d) Single Ad-CRISPRa-V transduced cells observed as HIV-1/GFP+ single positive cells [%] ±SD with n=3 from three independent experiments. Black bars (Untreated, TNF- α) show non-infected cells.


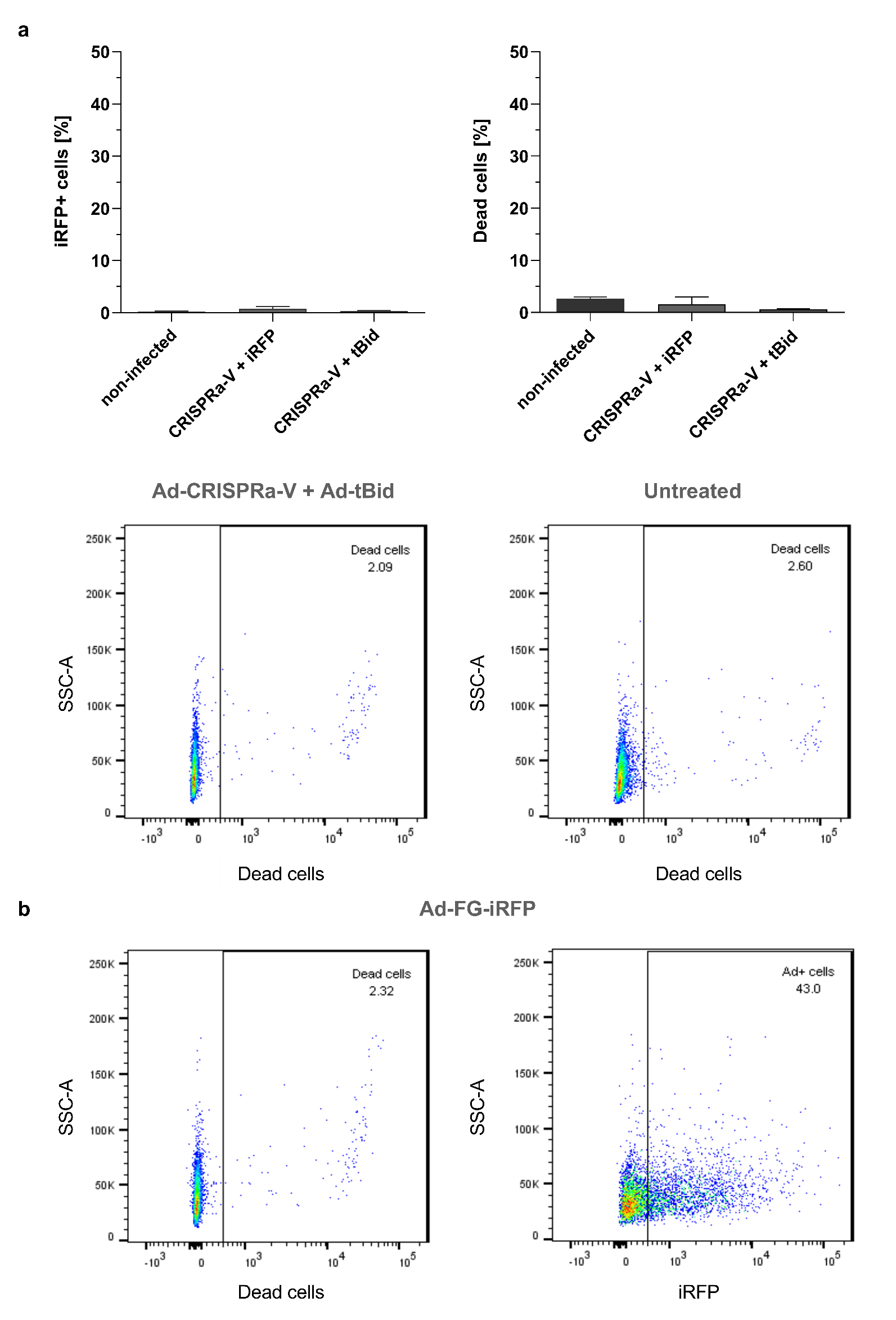


Supplementary figure S5: Ad-CRISPRa-V in combination with Ad-tBid or Ad-iRFP does not activate the expression of iRFP and tBid from the suicide vector**.** 1x10^5^ Jurkat cells were co-transduced with a total of 8x10^3^ VP/cell with CD3-retargeted Ad-CRISPRa-V and either Ad-tBid or Ad-iRFP. Ad-FG-iRFP control was transduced with a total of 4x10^3^ VP/cell. Ad coating was performed by preincubating Ads with CD3-retargeting adapters in a 50-fold molar excess over adenovirus fiber knob for 1.5 h on ice before addition to cells. HIV-1 latency reversal in the CRISPRa-Con and Cell controls was achieved by adding TNF-α [10 ng/ml] 24 h post transduction. At 48 h post-transduction cells were stained with the dead cell zombie dye and measured by flow cytometry. (a) Shown are iRFP transgene expression as iRFP+ cells [%] and cell death as dead cells [%] in co-transduced cells, ±SD with n=3 as well as exemplary flow cytometry plots. (b) Flow cytometry plots of Ad-FG-iRFP transduction control showing cell death and Ad transduced cell population as iRFP+ cells.
